# Case Report: Visceral Leishmaniasis with *Salmonella* Paratyphi and *Brucella melitensis* Coinfection as a Cause of Persistent Fever in a Patient from Sudan

**DOI:** 10.4269/ajtmh.18-0466

**Published:** 2018-09-24

**Authors:** Sayda El Safi, Hussam Elshikh, Enaam El Sanousi, Nagwa El Amin, Alfarazdag Mohammed, Kristien Verdonck, Jan Jacobs, Marleen Boelaert, François Chappuis

**Affiliations:** 1 Faculty of Medicine, University of Khartoum, Khartoum, Sudan;; 2 Brucella Department, Veterinary Research Institute, Khartoum, Sudan;; 3 Institute of Tropical Medicine, Antwerp, Belgium;; 4 Department of Microbiology and Immunology, KU Leuven, University of Leuven, Leuven, Belgium;; 5 Division of Tropical and Humanitarian Medicine, Geneva University Hospitals, Geneva, Switzerland

## Abstract

We describe the case of a 12-year-old boy from Sudan who presented with fever of 1-week duration, headache, cough, and vomiting. A set of diagnostic tests led to the diagnosis of three infectious diseases: visceral leishmaniasis (probable diagnosis based on positive direct agglutination test), enteric fever (blood culture grown with *Salmonella* Paratyphi), and brucellosis (blood culture grown with *Brucella melitensis*). The patient received specific treatment of the three infections and recovered. This case illustrates the occurrence and possible implications of coinfections in patients with persistent fever, including conditions that are hard to diagnose in field settings, such as brucellosis and enteric fever.

## CASE REPORT

A 12-year-old boy presented to the outpatient clinic of Tabarak Allah Rural Hospital in Gedaref State in October 2013, with complaints of fever, chills, headache, dry cough, and vomiting for 1 week, and with anorexia for the last 2 days. The patient had no history of visceral leishmaniasis, but he came from Barbar El Fugara village in the Atbara River area (lat. 13°34′47.7″N, long. 36°18′30.6″E), the most endemic area for visceral leishmaniasis in Sudan. He was enrolled in a clinical study called “Neglected Infectious Diseases Diagnosis” (NIDIAG)^1^ and underwent standard history taking, physical examination, and a set of diagnostic tests targeting severe and treatable infectious causes of persistent fever, that is, visceral leishmaniasis, malaria, tuberculosis, enteric fever, brucellosis, amebic liver abscess, relapsing fever, rickettsial diseases, leptospirosis, and human immunodeficiency virus (HIV) infection. The NIDIAG project did not interfere with the choice of treatment of the included patients, but made sure that essential medicines for the target conditions were present at the study site.

The initial physical examination (day 0) revealed that his weight was 21 kg, height 118 cm, axillary temperature 40.7°C, respiratory rate 30/minute, heart rate 108/minute, and blood pressure 90/70 mm Hg. He presented with a normal level of consciousness, moderate cachexia, pallor, cervical and inguinal lymphadenopathy (size 1 cm), and bilateral tonsil inflammation. Chest examination revealed crackles and decreased air entry in the right lung. No abnormalities were found on abdominal examination. The rest of the physical examination was unremarkable.

On laboratory testing, the hemoglobin level was 11.2 g/dL and the white blood cell count 12.6 × 10^9^/L. Urine analysis revealed 10–25 leukocytes/μL. Both Giemsa-stained blood microscopy and rapid diagnostic tests for malaria (Pf-HRP2 and pan-pLDH) were negative. A rapid diagnostic test for HIV (Determine^™^; Inverness Medical, Shinjuku-ku, Japan) was negative. Direct microscopical search for *Leishmania* amastigotes on lymph node aspirate was negative, and as the patient did not have clinical splenomegaly, spleen aspiration was not carried out. Blood samples were collected for the direct agglutination test for visceral leishmaniasis and for blood culture (using two HiMedia^™^ (HiMedia Laboratories, Mumbai, India) culture bottles and searching for *Salmonella* and *Brucella*).

On day 0, the clinical picture was consistent with bacterial pneumonia, and oral erythromycin and amoxicillin treatment was initiated. On day 2, as the high fever persisted, treatment was switched to intravenous ceftriaxone. The next day, the direct agglutination test was found to be positive (titer 1/12,800) and, accordingly, a diagnosis of probable visceral leishmaniasis was made. The patient was admitted to the hospital and received intramuscular sodium stibogluconate (20 mg/kg) combined with paromomycin (15 mg/kg) daily for 17 days, and ceftriaxone was stopped. The fever subsided in the following days and the patient was discharged on day 19 with no remaining symptoms and signs.

On day 12, the reference laboratory in Khartoum identified *Salmonella* Paratyphi in the blood culture taken on admission, but the result could only be communicated to the medical team in the field and subsequently to the patient on day 25. The paromomycin component of the treatment of visceral leishmaniasis could have had a partial effect on the patient’s salmonellosis, as paromomycin is an aminoglycoside with poor activity against intracellular bacteria. The attending physician decided to treat the patient with trimethoprim/sulfamethoxazole for 2 weeks. On day 43, *Brucella melitensis* biovar 1 was identified in the admission blood cultures and, despite the absence of symptoms, the patient was treated with oral doxycycline for 6 weeks and intramuscular gentamicin for 2 weeks. The patient remained well during treatment and follow-up.

## DISCUSSION

To our knowledge, this is the first report in the English literature of isolation of *Salmonella* and *Brucella* in blood cultures of a febrile patient with probable visceral leishmaniasis. The identification of *Salmonella* sp. and *Brucella* sp. at reference laboratories of the University of Khartoum was confirmed and further characterized as *Salmonella* Paratyphi (Microbiology Laboratory, Institute of Tropical Medicine, Antwerp, Belgium) and *B. melitensis* biovar 1 (Animal and Plant Health Agency, Surry, United Kingdom). Furthermore, molecular characterization of the *Brucella* isolate was performed at the Animal and Plant Health Agency using a multiplex polymerase chain reaction assay (Bruce-Ladder). The contribution of *Salmonella* Paratyphi and *B. melitensis* infection in the febrile presentation of the patient at admission is very likely as we are not aware of asymptomatic individuals with positive blood cultures with any of these two pathogens.

The diagnosis of visceral leishmaniasis was probable, given the high titer on direct agglutination testing. This positive serology result alone is insufficient for a definite diagnosis of visceral leishmaniasis because there is a background of seropositivity with the direct agglutination test and other serological methods among asymptomatic individuals living in endemic areas. The search for parasites in lymph nodes was negative, but this does not preclude the diagnosis of visceral leishmaniasis because of the limited sensitivity (around 50%) of this technique. The physical examination of our patient did not reveal spleen enlargement, but inguinal and cervical lymph nodes were found. Previous reports from Sudan indicated that 4% of visceral leishmaniasis patients did not present with splenomegaly^2^; the absence of splenomegaly, especially in a patient with only 1 week of fever, should therefore not be regarded as evidence that rules out a diagnosis of visceral leishmaniasis. Prolonged fever with lymphadenopathy should raise the suspicion of visceral leishmaniasis in Sudan.^3^

Half of the 30,000–50,000 annual visceral leishmaniasis cases estimated in East Africa, the second largest visceral leishmaniasis focus in the world after South Asia, are thought to occur in Sudan.^4^ In this country, several visceral leishmaniasis epidemics were reported, claiming a huge death toll.^5^ Gedaref State in the eastern part of the country is prone to epidemics and presently accounts for the vast majority of visceral leishmaniasis cases in Sudan, particularly in the Atbara River area.^6^

Although visceral leishmaniasis is a well-recognized and quantified health problem in Sudan, there are no such national data for enteric fever and brucellosis, and the exact contribution of these conditions to febrile illness in the country is not known. Enteric fever is a systemic illness caused by *Salmonella* Typhi and *Salmonella* Paratyphi (divided into three subtypes: A, B, and C) and widely distributed in Asia, Africa, and Latin America, with an estimated worldwide 21.6 million cases and 200,000 deaths in 2004.^7^ Transmission occurs through consumption of contaminated food and drinks handled by people who shed the organism from stool or, less commonly, urine. Classic presentation includes acute or persistent fever, abdominal pain, diarrhea or constipation, dry cough, and hepatosplenomegaly. Reports from Gedaref state indicate that 4,444 cases of enteric fever occurred in the state in 2010.^8^ This number is likely to be imprecise because of the lack of accurate diagnosis (i.e., blood culture) in most health facilities. Enteric fever was found to be the second most common laboratory-confirmed diagnosis for acute fever patients in a study conducted in Port Sudan in eastern Sudan.^9^

Brucellosis is one of the world’s most common zoonotic infections, with greater than half a million new cases annually and highly variable incidence rates ranging from 0.02 (France) to 269 per 100,000 per year (Iraq). It is caused by several species of *Brucella*, which are fastidious Gram-negative aerobic coccobacilli. Transmission to humans occurs mainly through contact with fluids from infected domestic animals such as cattle, goats, and sheep, or by ingestion of their unpasteurized milk and other dairy products.^10^ Brucellosis is a bacteremic systemic infection that may involve any organ of the body. The disease in humans presents as an acute or persistent febrile illness, with or without focal clinical symptoms and signs.^11^ Brucellosis is one of the important zoonotic diseases among livestock in Sudan, where several studies were conducted to investigate the prevalence of the infection among animals and the proportion of human contacts with positive serology.^12–14^ In addition, 29 patients with long-standing fever from Central Sudan were diagnosed with probable brucellosis on the basis of one serological test.^15^ Isolation of *Brucella abortus* in a patient originating from Gedaref Province was also reported in 1966.^16^

Comorbidity of visceral leishmaniasis with other nonspecific (e.g., pneumonia and meningitis) or specific infectious diseases including HIV/AIDS, tuberculosis, and malaria has been reported from different parts of the world including Sudan.^17–19^ Comorbidity of visceral leishmaniasis and salmonellosis was described in a 16-month-old baby with prolonged fever in Turkey.^20^ In that report, however, the diagnosis of enteric fever was based on serology (Widal test), and a false-positive result could not be ruled out. The occurrence of concurrent infections with *Salmonella* and *Brucella* in febrile illness has been reported in two patients with acute fever in Egypt, but the diagnosis of brucellosis was based on serology in both cases.^21^

Coinfections cause diagnostic and therapeutic challenges. This was well illustrated in the patient presented here. The relative contribution of the different conditions to the clinical symptoms and signs at admission, and the relative contribution of the different treatments to the patient’s recovery are impossible to disentangle. Although visceral leishmaniasis could be diagnosed on site because of the availability of serology (direct agglutination test), the results of *Salmonella* sp. and *Brucella* sp. could only be obtained and communicated to the caregivers, and ultimately to the patient, on days 25 and 43, respectively. In fact, these conditions would have been simply missed if blood cultures had not been implemented for the purpose of the NIDIAG study. We, therefore, highlight the need 1) to develop diagnostic guidance tools for persistent fever that consider that several conditions may coexist and 2) to develop and/or deploy field-based accurate diagnostic tests for both enteric fever and brucellosis.
